# Immunological persistence in 5 y olds previously vaccinated with hexavalent DTPa-HBV-IPV/Hib at 3, 5, and 11 months of age

**DOI:** 10.4161/21645515.2014.970494

**Published:** 2014-10-31

**Authors:** Sven A Silfverdal, Deepak Assudani, Sherine Kuriyakose, Olivier Van Der Meeren

**Affiliations:** 1 Department of Clinical Sciences; Pediatrics; Umeå University; Umeå, Sweden; 2 Vaccine Discovery and Development; GlaxoSmithKline Vaccines; Bangalore, India; Wavre, Belgium

**Keywords:** antibody persistence, booster, vaccine, vaccination schedule

## Abstract

The combined diphtheria-tetanus-acellular pertussis-hepatitis B-poliomyelitis/*Haemophilus influenza* vaccine (DTPa-HBV-IPV/Hib: *Infanrix*™ hexa, GlaxoSmithKline Vaccines) is used for primary vaccination of infants in a range of schedules world-wide. Antibody persistence after 4 DTPa-HBV-IPV/Hib doses in the first 2 y of life has been documented, but long-term persistence data following the 3, 5, 11–12 months (3–5–11) infant vaccination schedule, employed for example in Nordic countries, are limited. We assessed antibody persistence in 57 5-year-old children who had received either DTPa-HBV-IPV/Hib or DTPa-IPV/Hib (*Infanrix*™-IPV/Hib, GlaxoSmithKline Vaccines) in the 3–5–11 schedule. Among DTPa-HBV-IPV/Hib recipients, 7/12 retained seroprotective antibody concentrations for diphtheria, 10/12 for tetanus, 5/12 for hepatitis and 10/12 for Hib. Detectable antibodies were observed for 0/12 children for pertussis toxin (PT), 12/12 for filamentous haemagglutinin (FHA) and 8/12 for pertactin (PRN). Among DTPa-IPV/Hib recipients, 28/45 retained seroprotective anti-diphtheria concentrations, 34/44 for tetanus and 40/45 for Hib. Detectable antibodies were observed for 9/45 children for PT, 41/45 for FHA and 34/45 for PRN. Antibody persistence in DTPa-HBV-IPV/Hib and DTPa-IPV/Hib-vaccinees appeared similar in 5 y olds to that previously observed in children of a similar age who had received 4 prior doses of DTPa-HBV-IPV/Hib (or DTPa-IPV/Hib). As in subjects primed with 4 prior doses, we observed that antibodies markedly declined by 5 y of age, calling for the administration of a pre-school booster dose in order to ensure continued protection against pertussis.

## Abbreviations

μg/ml: micrograms per milliliter
CI: confidence interval
DTPa-HBV-IPV/Hib: - diphtheria-tetanus-acellular pertussis, hepatitis B, inactivated poliovirus and *Haemophilus influenzae* type b vaccine
DTPa-IPV/Hib: diphtheria-tetanus-acellular pertussis-inactivated poliovirus and *Haemophilus influenzae* type b vaccine
FHA: filamentous haemagglutinin
GMC: geometric mean antibody concentration
Hib: *Haemophilus influenzae* type b
HBs: anti-hepatitis B surface antigen
NA: not applicable
PRN: pertactin
PRP: polyribosylribitol phosphate
PT: pertussis toxin

### 

Infant vaccination against diphtheria, tetanus, pertussis, *Haemophilus influenzae* type b (Hib) and poliomyelitis is well established throughout Europe, with many countries also including primary vaccination against hepatitis B. The use of combination vaccines containing antigens targeting all 6 of these pathogens improves the timeliness of vaccination, and improves vaccine coverage rates compared to separate administration of multiple smaller combination or monovalent vaccines.^1,2^

In this study, we assessed the persistence of antibodies to all vaccine antigens in 5-year-old children who were vaccinated with the combined diphtheria-tetanus-acellular pertussis-hepatitis B-poliomyelitis-Hib vaccine (DTPa-HBV-IPV/Hib: *Infanrix™* hexa, GlaxoSmithKline Vaccines) in a 3, 5 and 11 month (3–5–11) schedule. DTPa-HBV-IPV/Hib has been in use in Europe since 2000. Most infant vaccination schedules in Europe recommend 3 primary vaccination doses with a booster dose administered during the second year of life. Sweden, Denmark, Iceland, Finland, Norway, Italy, Slovakia and Austria administer 2-dose primary vaccination with a booster at 11–12 months of age (3–5–11 schedule). Available evidence suggests that after the booster dose, the percentage of 3–5–11-primed children who achieve seroprotected/seropositive antibody concentrations against vaccine antigens is similar to that achieved by 4 vaccine doses.^3-5^

Extensive data demonstrate the immunogenicity and safety profile of DTPa-HBV-IPV/Hib in children from as young as 6 weeks of age when administered as a 4-dose schedule,^6^ or in the 3–5–11 schedule.^7^ While antibody persistence until the pre-school booster dose has been demonstrated in children who received 4 DTPa-HBV-IPV/Hib doses in infancy,^5,8^ studies evaluating persistence following administration of DTPa-HBV-IPV/Hib in the 3–5–11 month schedule have been limited to hepatitis B.^9-11^

Children in this study had received primary vaccination in study 105539 (www.clinicaltrials.gov NCT00307034), which was designed to assess the immunogenicity of pneumococcal conjugate vaccine containing 10 serogroups, administered in 3–5–11 schedule relative to a 4-dose schedule.^12^ Children in 4 countries were randomized (1:1) to receive pneumococcal conjugate vaccination according to either schedule. Children in Sweden and Slovakia received concomitant primary vaccination with DTPa-HBV-IPV/Hib and children in Norway and Denmark received concomitant DTPa-IPV/Hib. The concomitant primary vaccinations were administered at 3, 5 and 11–12 months of age in all children, according to national recommendations.

This Phase IV extension study (www.clinicatrials.gov NCT01358825) was conducted in 2 centers in Norway and 2 centers in Sweden between 30 May 2011 and 15 July 2011. A single blood sample (3.5ml) was collected from all children at 5 y of age. All children who had participated in the primary vaccination study in these countries (N = 200) were invited to enrol. Children could not participate if they had received DTPa, polio or hepatitis B vaccination (or had disease due to any of these pathogens) since the vaccination study, with the exception of hepatitis B vaccination in the DTPa-IPV/Hib group. Children initially vaccinated in Denmark and Slovakia had already received a preschool booster dose of DTP vaccine at the time of this study, and were therefore not eligible for follow-up of antibody persistence after primary vaccination. The study was approved by the Regional Committee for Medical Ethics Research - Region South West, Norway, and the Regional Ethical Review Board (Regionala etikprövningsnämnden) in Umeå, Sweden. The studies were conducted according to Good Clinical Practice and in accordance with the Declaration of Helsinki. Written informed consent was obtained from the parents/guardians of all subjects before study entry.

**Figure 1.**
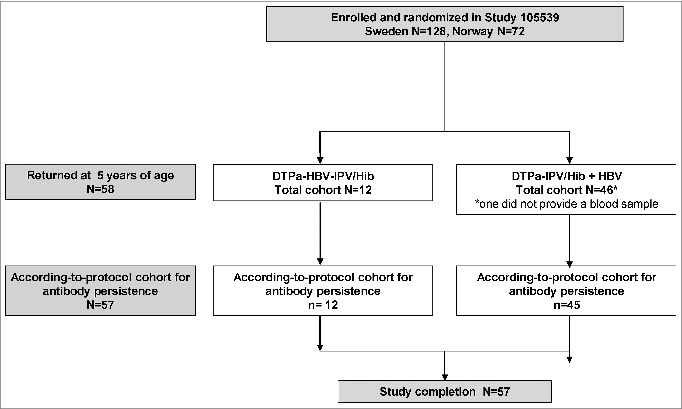
Subject disposition.

There were 58 children who returned at year 5, of whom 57 provided blood samples (**Fig. 1**). The according-to-protocol cohort for antibody persistence comprised 12 children in the DTPa-HBV-IPV/Hib group and 45 in the DTPa-IPV/Hib group. The most common reason for non-participation was ineligibility because the DTPa pre-school booster dose had already been administered, but also because some parents/guardians were unwilling to allow their child to participate. The mean age of all participating children was 5.0 y

.

Antibodies to polio types 1–3 were measured at GlaxoSmithKline's laboratories in Wavre, Belgium. Measurement of all other antibodies was done at GlaxoSmithKline's laboratories in Rixensart, Belgium (See Table 1 footnote). All tests were done using standardised, validated procedures with adequate controls and quality systems in place.

**Table 1:** Number* with antibody levels above assay cut-offs and geometric mean concentrations (GMC) in children 5 y of age after vaccination in infancy at 3, 5 and 11–12 months of age

		DTPa-HBV-IPV/Hib			DTPa-IPV/Hib		
		Post II	Post III	Persistence at 5 y of age	Post II	Post III	Persistence at 5 y of age
Diphtheria	≥0.1 IU/ml	11/11	11/11	7/12	37/38	39/39	28/45
	GMC (95% CI)	2.8 (1.4; 5.6)	8.8 (5.3; 14.9)	0.130 (0.1; 0.2)	0.9 (0.6; 1.4)	4.9 (3.8; 6.4)	0.2 (0.1; 0.3)
Tetanus	≥0.1 IU/ml	11/11	11/11	10/12	38/38	39/39	34/44
	GMC (95% CI)	4.2 (2.6; 6.7)	8.9 (6.3; 12.6)	0.3 (0.1; 0.6)	3.0 (2.2; 4.0)	9.9 (8.3; 11.9)	0.4 (0.2; 0.6)
PT	≥5 EU/ml	9/9	10/10	0/12	37/37	38/38	9/45
	GMC (95% CI)	32.3 (19.8; 52.7)	76.8 (50.5; 116.9)	2.5 (2.5; 2.5)	41.1 (32.6; 51.7)	89.9 (72.2; 112.0)	4.2 (2.9; 6.0)
FHA	≥5 EU/ml	9/9	10/10	12/12	37/37	38/38	41/45
	GMC (95% CI)	124.5 (62.5; 247.7)	325.8 (217.5; 488.1)	32.6 (11.5; 92.5)	208.9 (160.6; 271.8)	443.1 (345.4; 568.5)	50.7 (30.6; 84.1)
PRN	≥5 EU/ml	9/9	11/11	8/12	37/37	38/38	34/45
	GMC (95% CI)	105.3 (41.4; 268.0)	354.2 (193.6; 648.0)	6.2 (3.9; 9.9)	87.4 (58.9; 129.9)	265.5 (182.0; 387.2)	11.9 (8.4; 16.8)
HBV**	≥10 mIU/ml	6/6	7/7	5/12	NA	NA	NA
	GMC (95% CI)	249.8 (110.2; 566.2)	1436.6 (535.9; 3851.1)	9.0 (3.9; 20.9)	NA	NA	NA
Hib	≥0.15 μg/ml	9/10	11/11	10/12	35/38	39/39	40/45
	GMC (95% CI)	0.7 (0.2; 2.4)	16.5 (9.5; 28.5)	0.4 (0.2; 0.7)	1.5 (0.8; 2.7)	26.4 (17.3; 40.3)	0.9 (0.6; 1.4)

*The numerator is the number of children with antibody levels above the cut-off. The denominator is the number of children in the according-to-protocol cohort antibody persistence at each time point. NA = not applicable. CI = confidence intervals; Antibodies to diphtheria, tetanus, Hib and pertussis antigens were measured by ELISA with an assay cut-off of 0.1 IU/ml (D and T), 0.15 μg/ml (Hib) and 5 EL.U/ml (pertussis). Anti-HBs antibodies were measured at the post-2 and post-3 time points with an ELISA with a cut-off of 3.3 mIU/ml. Samples at 5 y of age were tested using an FDA approved and EU-marketed Chemiluminescence Immunoassay (CLIA) (Centaur™, Siemens, Germany) with a cut-off of 6.2 mIU/ml. Antibodies to D, T, pertussis antigens and Hib were measured using in-house assays as previously described.^9^

**While seroprotection rates are generally comparable between the anti-HBs assays, GMCs detected by the different assays may differ – indicated by [].

Among the children primed with DTPa-HBV-IPV/Hib, the number with seroprotective antibody concentrations at 5 y of age was 7/12 for diphtheria, 10/12 for tetanus, 5/12 for hepatitis B and 10/12 for Hib (Table 1). No child had anti-diphtheria antibody concentrations ≥1.0 IU/ml, 2/12 had anti-tetanus antibodies ≥1.0 IU/ml and 10/12 had antibodies for Hib ≥1.0 μg/ml. No child had detectable antibodies for pertussis toxin (PT), 12/12 were seropositive for anti-filamentous haemagglutinin (FHA) antibodies and 8/12 had anti-pertactin (PRN) antibodies.

Among children primed with DTPa-IPV/Hib, the number with seroprotective antibody concentrations was 28/45 for diphtheria, 34/44 for tetanus, and 40/45 for Hib. The number of children with diphtheria and tetanus antibody concentrations ≥ 1.0 IU/ml was 6/45 and 6/44, respectively, and there were 40/45 children with antibodies for Hib ≥1.0 μg/ml. There were 9/45 children with detectable anti-PT antibodies, 41/45 with anti-FHA and 34/45 with anti-PRN antibodies.

All antibody geometric mean concentrations (GMCs) had markedly decreased at 5 y of age compared to post-dose-3 levels (**Table 1**).

Results of poliovirus testing using a micro-neutralization assay were invalid due to unresolved technical issues.

Antibody GMCs after the second primary vaccination and after the booster at 11–12 months of age were calculated in children who did, and who did not participate in the persistence study in order to assess whether selection bias had occurred. The antibody GMCs after dose 2 and 3 administered in the primary vaccination study were within the same range (95% confidence intervals overlapped) in participating children and in the cohort of non-participating children, with the exception of antibodies to PT, which were observed to be higher in children in the DTPa-IPV/Hib group who participated in the persistence study. A sensitivity analysis assessed the impact of the high subject drop-out on the results at year 5. This analysis took into account the antibody concentrations observed in serum samples collected after dose-3 in the previous vaccination study. The model indicated that the analysis was not unduly affected by subjects who dropped out.

This study is limited by its small sample size, predominantly because the scheduled booster dose had already been administered, making most children ineligible for follow-up. Statistical comparisons were thus not done, and while firm conclusions cannot be drawn because of the small number of participating children, several observations are noteworthy. The persistence of antibodies for antigens common to DTPa-HBV-IPV/Hib and DTPa-IPV/Hib appeared to be similar between both groups. Seroprotection/seropositivity for antibodies to diphtheria, tetanus and pertussis vaccine antigens in DTPa-HBV-IPV/Hib and DTPa-IPV/Hib vaccinees at year 5 was in the range reported in cohorts of children of a similar age who had received 4 prior DTPa-HBV-IPV/Hib doses.^5,8^ Persistence of antibodies to hepatitis B and Hib appeared lower in study participants than in children who had received 4 previous doses. However, for hepatitis B it is generally accepted that robust anamnestic responses rather than persisting antibodies are necessary for protection.^13,14^ Two studies assessed anamnestic response to hepatitis B in children primed with DTPa-HBV-IPV/Hib at 3, 5 and 11–12 months of age. Five years after vaccination of Italian children with DTPa-HBV-IPV/Hib, 83.2% children continued to have anti-hepatitis B surface antigen (HBs) antibodies ≥10 mIU/ml.^11^ A challenge dose of hepatitis B vaccine induced seroprotective titres (≥10 mIU/ml) in 94.3% of children.^11^ Robust immune memory responses to hepatitis B were also observed in children 10–11 y after vaccination with DTPa-HBV-IPV/Hib at 3, 5 and 11–12 months of age, with 96.8% of children achieving an anamnestic response (4-fold increase in anti-HBs antibody concentration in initially seropositive children, or concentration ≥10mIU/ml in initially seronegative children) after the challenge dose.^9^ These results compare favorably to studies of immune memory after 4 doses of HBV vaccine in infancy, administered either as DTPa-HBV-IPV/Hib or as monovalent HBV.^15,16^

Based on assessment of Hib vaccine failures in the United Kingdom, protection against Hib disease is thought to be mediated by circulating antibody, with immune memory responses too slow to prevent invasive disease.^17^ In our study, the majority of children in both groups continued to have anti-PRP antibodies ≥0.15 μg/ml at 5 y of age, after which time the incidence and severity of invasive Hib disease declines dramatically.^18^ Thus results of our study suggest that while persisting anti-PRP concentrations in 5 y old children are lower after 3 doses in infancy than after 4 doses, protection after 3 doses is sufficient to last until the age when invasive Hib disease is no longer a major threat. Serological surveillance data from Sweden support that Hib disease remains well controlled without additional Hib boosters after 12 months of age.^19^

Low levels of detectable anti-PT antibodies in both groups at 5 y of age suggest little exposure to pertussis in these children, but susceptibility to infection, and support the administration of a pre-school booster.

In conclusion, antibody persistence was observed for diphtheria, tetanus, hepatitis B, Hib, FHA and PRN in 5-year-old children after priming with DTPa-HBV-IPV/Hib at 3, 5 and 11–12 months of age. While our study is limited by the small number of children who returned for follow-up, a comparison of observed versus expected antibody persistence using longitudinal modeling suggests that the results were indicative of the larger cohort. As in subjects primed with 4 prior doses, we observed that antibodies markedly declined by 5 y of age, calling for the administration of a pre-school booster dose by 4 to 5 y of age in order to ensure continued protection against pertussis.

INFANRIX is a trademark of the GlaxoSmithKline group of companies. CENTAUR is a trademark of Siemens, Germany.
